# Footsteps to Wellness: A Systematic Review and Meta-Analysis of Walking Pace and Coronary Artery Disease Event

**DOI:** 10.7759/cureus.56926

**Published:** 2024-03-25

**Authors:** Yusuf Aji S Nurrobi, Kevin Winston, Andi L Rahman, Moh F Falakhi, Meutia P Aristya, Ahmad F Toaha, Iva N Larasaty, Raditya Dewangga

**Affiliations:** 1 Cardiology and Vascular Medicine, Pertamina Hospital, Balikpapan, IDN; 2 Hospital Medicine, Bhakti Medicare Hospital, Cicurug, IDN; 3 General Medicine, Hasri Ainun Habibie Regional Hospital, Parepare, IDN; 4 General Medicine, Muhammadiyah Gresik Hospital, Gresik, IDN; 5 General Medicine, Metropolitan Medical Centre Hospital, Jakarta, IDN; 6 General Medicine, Labuang Baji Hospital, Makassar, IDN; 7 General Medicine, Halu Oleo University, Kendari, IDN; 8 Emergency Medicine, Rumah Sakit Umum Daerah (RSUD) Gunung Jati, Cirebon, IDN

**Keywords:** cardiology, review, prevention, coronary artery disease, walking pace

## Abstract

Coronary artery disease (CAD) poses a global health challenge, necessitating effective preventive strategies. Despite the pivotal role of physical activity in cardiovascular health, many fall short of recommended guidelines for daily physical activity. Simple and accessible, walking presents an opportunity, with increased pace emerging as a potential strategy for reducing the risk of cardiovascular diseases. Thus, we aimed to elucidate the potential association between walking pace and the risk of CAD events in adults without a prior history of CAD through a systematic review. We searched PubMed, Scopus, Web of Science, and ScienceDirect without publication date restrictions to identify prospective cohorts that analyzed walking pace and adult CAD events. The literature search conducted from April 02, 2023, to August 21, 2023, identified a total of four studies (six cohorts) for meta-analysis using random-effects models. The Newcastle-Ottawa Scale was used to assess study quality, and data extraction involved two independent reviewers. The analysis calculated overall relative risks (RRs) and 95% confidence intervals (CIs) for those with the quickest walking paces compared to those with the slowest walking paces. A funnel plot analysis for publication bias and subgroup analysis were also conducted. Results from the meta-analysis involving 160,519 participants and 3,351 CAD events demonstrated a 46% decreased risk for those walking at the quickest pace (pooled RR = 0.54, 95% CI = 0.45-0.66). No significant heterogeneity was observed. In conclusion, walking pace emerges as a significant risk factor for CAD events in adults without a prior history of CAD. It serves as a potential screening tool to identify individuals at higher risk. Promoting a faster walking pace as a daily activity may effectively mitigate the burden of CAD.

## Introduction and background

Coronary artery disease (CAD) is an important and prevalent global health concern, constituting a substantial burden on public health systems. It is one of the major global causes of morbidity and mortality across the globe [1]. Despite significant advancements in our understanding of its risk factors and preventive measures, CAD continues to remain a challenge for healthcare professionals and researchers. Physical activity has long been recognized as a cornerstone of cardiovascular health. The benefits of regular exercise in preventing CAD and improving overall cardiovascular well-being are firmly established [2,3]. However, a disconcerting reality persists - a substantial proportion of the global population fails to achieve recommended physical activity guidelines [4-6]. For example, approximately 50% of U.S. citizens do not adhere to the recommended physical activity guidelines [6]. Additionally, in an increasingly urbanized world, more and more people do not have the necessary time to perform physical activities [7]. The lack of physical activity constitutes a significant public health concern, contributing to the increasing prevalence of CAD, cancer, and metabolic disorders such as diabetes and hypercholesterolemia [7].

In 2020, the World Health Organization (WHO) unveiled global physical activity guidelines, underscoring the importance of regular exercise in reducing the risk of non-communicable diseases, including CAD [8]. While these guidelines serve as a roadmap for health promotion, there remains a considerable gap between knowledge and implementation. Barriers to achieving recommended physical activity levels are multifaceted, including issues related to motivation, access, perception, and the prescription of sustainable physical activity by healthcare professionals [4].

In this context, the notion of walking, a fundamental human activity, takes center stage. Walking is remarkably simple, requiring no special skills or equipment, and is accessible across age groups [9]. It offers a low-risk approach to promoting physical activity. Importantly, the concept of walking pace emerges as a relatively unexplored aspect of this ubiquitous activity.

The significance of enhancing cardiovascular health through walking pace lies in its potential as an easily accessible and straightforward strategy. By incorporating the element of speed into a daily activity as commonplace as walking, the risk of CAD and its associated health and economic burdens can be reduced. Despite promising signals from prospective cohort studies that suggest a potential correlation between a faster walking pace and reduced CAD risk, a comprehensive understanding of this relationship is lacking.

This study aims to bridge this knowledge gap by investigating the association between walking pace and the risk of CAD, with a specific focus on adults who have not previously experienced CAD. This study also aims to provide valuable insights that could inform public health strategies and clinical recommendations for cardiovascular disease prevention, with a particular emphasis on a universally accessible, yet underappreciated, form of physical activity.

## Review

Methodology

Search Strategy

This study followed the guidelines as outlined in the Preferred Reporting Items for Systematic Reviews and Meta-Analyses (PRISMA) [10]. We performed a thorough search for relevant prospective cohort studies in key databases, including PubMed, Web of Science, Scopus, and ScienceDirect. To ensure comprehensiveness, we also performed additional searches on Google and scrutinized citations within identified studies. The search strategy encompassed specific keywords, such as “walking pace,” “coronary artery disease,” “myocardial infarction,” “cohort,” and “follow up.” The search strategy is presented in Table 1.

**Table 1:** Details of the search strategy.

No	Database	Search strategy
1	PubMed	(“walking speed”[Title/Abstract] OR “gait”[Title/Abstract] OR “walking pace”[Title/Abstract] OR “gait speed”[Title/Abstract] OR “walking velocity”[Title/Abstract] OR “ambulation speed”[Title/Abstract] OR “gait velocity”[Title/Abstract] OR “walking speed”[MeSH Terms]) AND (“coronary artery disease”[Title/Abstract] OR “myocardial infarction”[Title/Abstract] OR “coronary heart disease”[Title/Abstract] OR “ischaemic heart disease”[Title/Abstract] OR “ischemic heart disease”[Title/Abstract] OR “atherosclerotic heart disease”[Title/Abstract] OR “coronary atherosclerosis”[Title/Abstract] OR “cad”[Title/Abstract] OR “chd”[Title/Abstract] OR “coronary artery disease”[MeSH Terms] OR “myocardial infarction”[MeSH Terms] OR “acute coronary syndrome”[MeSH Terms]) AND (“cohort”[All Fields] OR “prospective”[All Fields] OR “follow up”[All Fields])
2	Scopus	(TITLE-ABS-KEY(“coronary artery disease”) OR TITLE-ABS-KEY(“coronary heart disease”) OR TITLE-ABS-KEY(“myocardial infarction”) OR TITLE-ABS-KEY(“ischaemic heart disease”) OR TITLE-ABS-KEY(“ischemic heart disease”) OR TITLE-ABS-KEY(“atherosclerotic heart disease”) OR TITLE-ABS-KEY(“coronary atherosclerosis”) OR TITLE-ABS-KEY(“cad”) OR TITLE-ABS-KEY(“chd”) OR TITLE-ABS-KEY(“acute coronary syndrome”)) AND (TITLE-ABS-KEY(“walking speed”) OR TITLE-ABS-KEY(“walking pace”) OR TITLE-ABS-KEY(“gait speed”) OR TITLE-ABS-KEY(“walking velocity”) OR TITLE-ABS-KEY(“gait velocity”) OR TITLE-ABS-KEY(“ambulation speed”)) AND (ALL(“cohort”) OR ALL(“prospective”) OR ALL(“follow up”))
3	Web of Science	(TS=(“walking speed” OR “gait” OR “walking pace” OR “gait speed” OR “walking velocity” OR “ambulation speed” OR “gait velocity”)) AND (TS=(“coronary artery disease” OR “myocardial infarction” OR “coronary heart disease” OR “ischaemic heart disease” OR “ischemic heart disease” OR “atherosclerotic heart disease” OR “coronary atherosclerosis” OR “cad” OR “chd” OR “acute coronary syndrome”)) AND (TS=(“cohort” OR “prospective” OR “follow up”))
4	ScienceDirect	(“walking speed” OR “walking pace”) AND (“coronary artery disease” OR “myocardial infarction” OR “coronary heart disease” OR “acute coronary syndrome”) AND (“cohort” OR “prospective” OR “follow up”)

Eligibility Criteria and Study Selection

Two authors (YASN and KW) individually performed the comprehensive screening, which encompassed the evaluation of titles, abstracts, and full-text articles. Disparities were resolved through consensus with all authors. The selection criteria for the studies encompassed the following aspects: studies should employ a prospective cohort design, involve adults without a prior CAD history, and provide information on relative risks (RRs) along with their 95% confidence intervals (CIs) in connection to CAD and walking pace. Additionally, the selected studies needed to be published in English. These assessments were considered only if they had at least controlled for age as a confounding factor or if they provided enough data to calculate such statistics.

Exclusion criteria encompassed the following conditions: studies not adhering to the prospective cohort design, studies involving children or adolescents, instances where the outcome (CAD) was present at baseline, and studies not published in English.

Outcome Definition

We defined CAD events as both fatal and non-fatal myocardial infarction in accordance with the criteria specified in the included studies.

Data Extraction

Two authors (YASN and KW) individually performed the data extraction. Disparities were resolved through consensus from all authors. The following information was extracted from the articles: last name of the first author, study year, study location, baseline age, gender distribution, follow-up period, participant count, CAD event count, walking pace assessment methods, CAD outcome ascertainment, walking pace categories, adjusted RRs with 95% CIs, and covariates adjusted in the final model.

Quality Assessment

To assess the quality of the included prospective cohort studies, the Newcastle-Ottawa Scale (NOS) was utilized, which is a recognized and established tool known for its systematic evaluation of non-randomized research [11]. NOS consists of three categorical criteria and a maximum achievable score of 9 points, a scoring framework established in earlier research [12]. Studies receiving a score of 7 points or more were categorized as “good,” while those scoring between 2 and 6 points were classified as “fair.” Studies with a score of 1 point or less were designated as “poor” in quality. To enhance the reliability of this review, we exclusively incorporated primary studies falling within the “fair” to “good” quality range.

Statistical Analysis

Within our statistical analysis, the initial focus was on exploring the link between walking pace and the risk of CAD by comparing individuals in the fastest walking pace category with those in the slowest category. Employing a random-effects model, we calculated pooled RRs and their corresponding 95% CIs. The inclusion criteria comprised studies that effectively addressed numerous potential confounding variables in their analyses.

We conducted stratified analyses to explore potential effect modifications based on predefined factors. These factors included gender (males and females), study population (>40,000 participants and ≤40,000 participants), baseline age (>55 years and ≤55 years), and follow-up period (>8 years and ≤8 years). To appraise the variability across studies, the Cochran Q test was employed, and the quantification was done using the I^2^ statistic [13]. A significance level of p < 0.1 was used for the Cochran Q test. We categorized I^2^ values into none, low, moderate, or high levels of heterogeneity, corresponding to values below 25%, between 25% and 50%, between 50% and 75%, and 75% or greater, respectively. Visual inspection of funnel plots was performed to investigate potential publication bias. We conducted all analyses using Review Manager (RevMan) software version 5.4. Statistical significance was set at a two-sided p-value below 0.05.

Results

Identification and Study Selection

A thorough search of several databases, including PubMed, Web of Science, Scopus, and ScienceDirect, revealed a substantial collection of 679 studies (95 from PubMed, 422 from Scopus, 151 from Web of Science, and 11 from ScienceDirect). Of these, 159 studies were excluded due to being duplicates, and one study was found to be retracted, which left 519 studies for screening.

**Figure 1:**
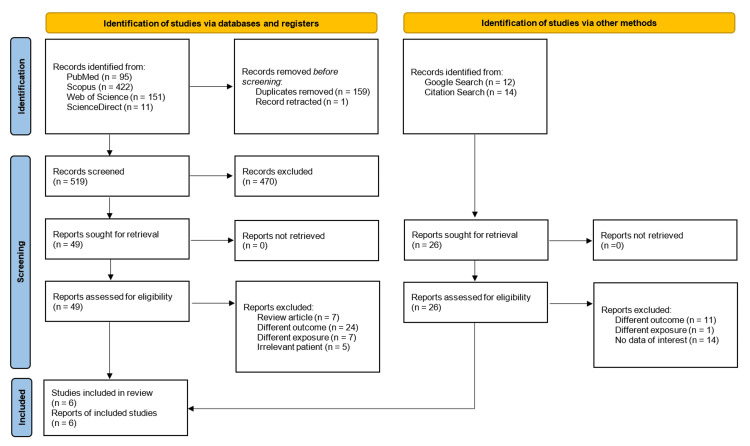
Study selection process (Preferred Reporting Items for Systematic Reviews and Meta-Analyses flow diagram).

Subsequent screening excluded 470 studies that were deemed irrelevant upon abstract assessment, leaving 49 studies for full-text assessment. From 49 studies, we further excluded seven non-original studies (e.g., review articles), 24 studies with disparate outcomes, seven studies exploring different exposures, and five studies with populations that did not align with our research focus. A total of 26 studies were identified from Google search and manual citation search but all of them were found to be unsuitable to be included in review. Overall, the literature search resulted in a total of six studies for inclusion in this systematic review and meta-analyses (Figure 1).

Study Characteristics

The dataset for our systematic review included six studies spanning from 1999 to 2022 (Table 2) [14-19]. These studies involved a total of 543,584 participants with an average age of 56.7 years, including 40.2% males. We observed 14,258 events of CAD across these studies. Geographically, four studies were from the United States [15,16,18,19], one study from Japan [14], and one study from the United Kingdom [17].

**Table 2:** Systematic review table of walking pace and the risk of CAD. CAD = coronary artery disease; CI = confidence interval; RR = relative risk

Author’s name	Year	Location	Baseline age	Gender	Follow-up period	Participants	Events	Walking pace assessment	Outcome ascertainment	Walking pace categories (km/h)	RR (95% CI)	Adjusted variables
Soares-Miranda et al. [15].	2016	United States	Mean: 72.5	Mixed	Maximum: 10 years	4,207	762	Self-reported	Medical records	<3.2 km/h, 3.2–4.7 km/h, ≥4.8 km/h	1 (reference) 0.66 (0.56–0.78), 0.50 (0.38–0.67)	Age, body mass index, clinical sites, education, income, race, sex, smoking
Tanasescu et al. [18].	2002	United States	Median: 57.5	Male	Maximum: 12 years	44,452	1,700	Self-reported	Medical records	<3.2 km/h, 3.2–4.7 km/h, 4.8–6.3 km/h, ≥6.4 km/h	1 (reference) 0.74 (0.60–0.91), 0.60 (0.45–0.79), 0.50 (0.30–0.83)	Alcohol intake, cholesterol, diabetes, hypertension, nutrition intake, parental history of myocardial infarction, smoking
Lee et al. [19].	2001	United States	Mean: 53.9	Female	Mean: 7 years	39,372	244	Self-reported	Medical records	Not walking regularly, <3.2 km/h, 3.2–4.7 km/h, ≥4.8 km/h	1 (reference) 0.56 (0.32–0.97), 0.71 (0.47–1.05), 0.52 (0.30–0.90)	Age, alcohol, fruit and vegetable consumption, menopausal status, parental history of myocardial infarction, postmenopausal hormone use, randomized treatment assignment, saturated fat, smoking
Manson et al. [16]	1999	United States	Mean: 52.2	Female	Maximum: 8 years	72,488	645	Self-reported	Medical records	<3.2 km/h, 3.2–4.7 km/h, ≥4.8 km/h	1 (reference) 0.75 (0.59–0.96), 0.64 (0.47–0.88)	Age, alcohol intake, body mass index, history of hypertension, menopausal status, parental history of myocardial infarction, smoking, study period, vitamin supplement use
Klinpudtan et al. [14]	2021	Japan	Mean: 76	Mixed	Maximum: 7 years	1,272	45	Timed walking pace test	Self-reported	<3.6 km/h, >3.6 km/h	1 (reference) 0.38 (0.18-0.78)	Nil
Zaccardi et al. [17]	2022	United Kingdom	Mean: 57.5	Mixed	Median: 12 years	380,693	10,862	Self-reported	Medical records	<4.8 km/h, 4.8–6.3 km/h. ≥6.4 km/h	1 (reference) 0.63 (0.59–0.66), 0.40 (0.38–0.43)	Nil

The study population varied significantly, ranging from 1,272 to 380,693 participants, with follow-up periods spanning 7 to 12 years. Study populations comprised three studies with both genders [14,15,17], two studies with only female participants [16,19], and one study with solely male participants [18]. CAD outcomes were ascertained through medical records in five studies [15-19], and via self-reporting in one study [18].

In terms of assessing walking pace, five studies used self-reported questionnaires [15-19] and one study employed a timed walking pace test [18]. The reporting of walking speed varied, with four studies presenting it in miles per hour (mph) [15-18], one study in kilometers per hour (km/h) [19], and one study in meters per second (m/s) [14]. Conversion factors were applied for uniformity. The conversion from miles per hour (mph) to kilometers per hour (km/h) was accomplished by multiplying mph values by 1.609. Similarly, the conversion from meters per second (m/s) to kilometers per hour (km/h) was executed by multiplying m/s values by 3.6.

Of the studies, two were excluded from the meta-analysis because they provided unadjusted data and conducted univariate analyses [14,17]. All four selected studies were included in a categorical association analysis, comparing CAD risk between the fastest and slowest walking pace categories, based on each study’s criteria. This analysis encompassed four studies that contributed to five cohorts. The reason behind this is that one study had a mixed-gender population. To accommodate this mixed-gender study, the data were separated into two distinct cohorts, one for males and one for females. Consequently, our analysis involved a total of five cohorts, comprising 160,519 individuals, with an average age of 54.6 years, including 28% male participants, and documented a total of 3,351 CAD events.

The quality assessment result is presented in Table 3. Overall, all studies were assessed as good quality based on NOS.

**Table 3:** Quality assessment of the included studies using the Newcastle-Ottawa Scale.

Study	Selection	Comparability	Outcome	Score
Soares-Miranda et al., 2016 [15]	3	2	3	8
Tanasescu et al., 2002 [18]	2	2	3	7
Lee et al., 2001 [19]	2	2	3	7
Manson et al., 1999 [16]	2	2	3	7
Klinpudtan et al., 2021 [14]	3	2	2	7
Zaccardi et al., 2022 [17]	3	2	3	8

Walking Pace and CAD Risk

**Figure 2:**
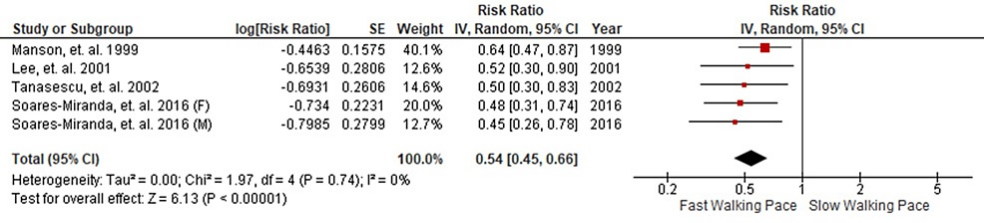
Forest plot comparing fastest versus slowest walking paces and CAD event risk. CAD = coronary artery disease

A random-effects model was applied to compare the fastest and slowest walking paces across a prospective cohort comprising 160,519 patients. This analysis revealed a 46% reduction in CAD event risk among participants in the fastest walking pace group. The RR was calculated at 0.54, and the 95% CI ranged from 0.45 to 0.66. It is pertinent to note the absence of statistically significant heterogeneity, as denoted by a p-value of 0.74 and an I^2^ value of 0% (Figure 2).

Publication Bias

**Figure 3:**
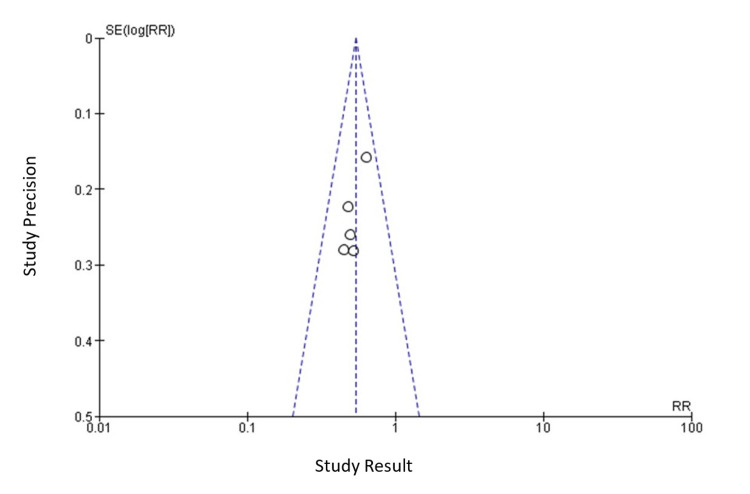
Funnel plot for publication bias analysis.

A funnel plot was created using Revman 5.4 (Figure 3). Due to the small number of studies used for the funnel plot, the possibility of publication bias cannot be eliminated as it is difficult to assess the funnel plot.

Subgroup Analysis

When assessing the fastest versus slowest walking pace categories, the noted inverse relationships between walking speed and CAD risk remained relatively consistent, irrespective of gender, participant count, follow-up period, and baseline age (Table 4).

**Table 4:** Subgroup analysis of included studies. CI = confidence interval; RR = relative risk

Modifiers	Subgroups	Σ Cohorts	RR (95% CI)	P-Heterogeneity	I^2^
All studies	-	5	0.54 (0.45–0.66)	0.74	0%
Gender	Male	2	0.48 (0.33–0.69)	0.78	0%
	Female	3	0.57 (0.45–0.72)	0.54	0%
Follow-up	>8 years	3	0.48 (0.36–0.63)	0.96	0%
	<8 years	2	0.61 (0.47–0.80)	0.52	0%
Participants	>40,000	2	0.60 (0.46–0.78)	0.42	0%
	<40,000	3	0.48 (0.36–0.64)	0.94	0%
Baseline age	>55 years	3	0.48 (0.36–0.63)	0.96	0%
	<55 years	2	0.61 (0.47–0.80)	0.52	0%

Discussion

Interpretation of Findings

This study employed a random-effects model to investigate the association between walking pace and the risk of CAD across a prospective cohort of 160,519 patients. Our analysis unveiled a substantial 46% reduction in CAD risk among individuals in the fastest walking pace category. This effect was quantified by an RR of 0.54, with a 95% CI of 0.45 to 0.66. Notably, the absence of statistically significant heterogeneity, indicated by a p-value of 0.74 and an I^2^ value of 0%, underscored the consistency and robustness of our findings. Additionally, a funnel plot (Figure 3) generated using RevMan 5.4 confirmed the absence of publication bias, further enhancing the credibility of our results. Importantly, the inverse relationship between walking speed and CAD risk was observed consistently, with a 39-52% reduction in CAD risk, irrespective of gender, participant count, follow-up period, and baseline age (Table 2).

Other Benefits of Walking Faster

A faster walking pace is also linked to various health benefits. A faster walking pace has been linked to a substantial 64% reduction in the likelihood of cognitive decline [20]. Additionally, individuals with slower gait speed have shown an increased likelihood of experiencing elevated depressive symptoms, heightened anxiety symptoms, and cognitive impairment [21]. Moreover, a slow walking pace is linked to a significantly increased risk of all-cause mortality, denoted by a hazard ratio (HR) of 1.89, indicating a nearly twofold higher risk of mortality [22]. These findings underscore the diverse health advantages of faster walking, including improved cognitive health, mental well-being, and increased longevity.

The Role of Behavioral Science

To establish faster walking as a sustainable daily habit, a deep comprehension of behavioral science is paramount. Within the framework of the Fogg Behavior Model, introduced by BJ Fogg, behavioral patterns are determined by three fundamental factors, namely, motivation, ability, and triggers. These elements collectively play a pivotal role in the success of habit formation [23]. Notably, the ease of habit execution, particularly during phases of diminished motivation, is of utmost significance.

In our opinion, walking, compared to structured daily exercises, is a more feasible and adaptable habit due to its accessibility. Moreover, habit adoption and permanence require a seamless integration into daily routines. In this context, incorporating faster walking can be seamlessly linked to existing daily habits. For instance, as individuals arrive at their workplace each day, initiating a brisk walk can become an integral part of their morning routine, ensuring a consistent and enduring incorporation of this health-enhancing habit.

Another fundamental concept in behavior science, as elucidated by Charles Duhigg, is the habit loop. This loop comprises three integral stages, namely, cue, response, and reward [24]. To illustrate, consider the act of incorporating faster walking as a newfound daily habit. The cue, in this instance, could be the moment of arriving at one’s workplace. This act serves as the signal that initiates the response, which involves engaging in brisk walking. The reward, a crucial component of habit formation, can take the form of a comforting cup of tea to start the day. By explicitly connecting the arrival at the workplace with the act of walking at a faster pace, individuals are not only enhancing their physical well-being but also harnessing the inherent power of behavioral science to facilitate the integration of this health-boosting practice into their daily lives.

While it has been shown that a faster walking pace is important to cardiovascular health, it is acknowledged that encouraging society to adopt a faster walking pace or simply to walk more often is difficult. One of the hurdles in this endeavor is the widespread absence of pedestrian-friendly zones, a concern that is notably pronounced in many developing countries [25]. This deficiency often prompts people to opt for vehicular transportation instead of walking, thereby impeding the adoption of fast walking.

It is also important to note that walking in a polluted environment predisposes to health hazards from pollution which may negate the beneficial effect of walking. All of the included studies in our review were conducted in developed countries, namely the United States, which tend to have better air quality than developing countries. This means that there is a possibility that the faster walking pace effect is attenuated in developing countries.

Study strengths and limitations

This study possesses several key strengths by leveraging prospective cohort studies, renowned for mitigating selection and recall biases, which are essential for long-term tracking of walking pace, a feasibility challenge in randomized controlled trials ideal for establishing causation. Additionally, the inclusion of studies with large sample sizes and long follow-up durations enhances the robustness of our findings. Third, the absence of heterogeneity adds to the robustness of the findings.

However, the study also presents certain limitations. Several cohorts relied on self-reported assessments of walking pace and self-reported outcome ascertainment. This introduces the potential for individual perception biases. Additionally, several included articles primarily focused on specific populations, such as nurses and medical professionals, rather than a more general representation of the population. This may limit the broader applicability of the findings. This study also restricted the literature search to publications available only in the English language, which may limit the articles selected. Lastly, this study lacks a dose-response analysis, which would have provided a more in-depth understanding of the association between walking pace and CAD risk.

## Conclusions

This study highlights a strong association between walking pace and prevention of CAD, with a 46% lower risk for fast walking pace. Promoting brisk walking as a daily habit offers a cost-effective means to improve heart health and has additional benefits for cognition, mental well-being, and longevity. Leveraging behavioral science can seamlessly integrate brisk walking into daily routines. The implications are significant. Brisk walking can serve as a simple tool to identify individuals at a higher risk of CAD and potentially reduce the global CAD burden.

## References

[1] (2024). The top 10 causes of death. https://www.who.int/news-room/fact-sheets/detail/the-top-10-causes-of-death.

[2] Nystoriak MA, Bhatnagar A (2018). Cardiovascular effects and benefits of exercise. Front Cardiovasc Med.

[3] Myers J (2003). Cardiology patient pages. Exercise and cardiovascular health. Circulation.

[4] Herazo-Beltrán Y, Pinillos Y, Vidarte J, Crissien E, Suarez D, García R (2017). Predictors of perceived barriers to physical activity in the general adult population: a cross-sectional study. Braz J Phys Ther.

[5] Elgaddal N, Kramarow EA, Reuben C (2022). Physical activity among adults aged 18 and over: United States, 2020. NCHS Data Brief.

[6] Tsao CW, Aday AW, Almarzooq ZI (2023). Heart disease and stroke statistics-2023 update: a report from the American Heart Association. Circulation.

[7] Park JH, Moon JH, Kim HJ, Kong MH, Oh YH (2020). Sedentary lifestyle: overview of updated evidence of potential health risks. Korean J Fam Med.

[8] Bull FC, Al-Ansari SS, Biddle S (2020). World Health Organization 2020 guidelines on physical activity and sedentary behaviour. Br J Sports Med.

[9] Murtagh EM, Murphy MH, Boone-Heinonen J (2010). Walking: the first steps in cardiovascular disease prevention. Curr Opin Cardiol.

[10] Moher D, Liberati A, Tetzlaff J, Altman DG (2009). Preferred reporting items for systematic reviews and meta-analyses: the PRISMA statement. PLoS Med.

[11] Wells G, Shea B, O’Connell D (2024). The Newcastle-Ottawa Scale (NOS) for assessing the quality of nonrandomised studies in meta-analyses. http://www.ohri.ca/programs/clinical_epidemiology/oxford.asp.

[12] Mengist B, Desta M, Tura AK (2021). Maternal near miss in Ethiopia: protective role of antenatal care and disparity in socioeconomic inequities: a systematic review and meta-analysis. Int J Afr Nurs Sci.

[13] Higgins JP, Thompson SG (2002). Quantifying heterogeneity in a meta-analysis. Stat Med.

[14] Klinpudtan N, Kabayama M, Godai K (2021). Association between physical function and onset of coronary heart disease in a cohort of community-dwelling older populations: the SONIC study. Arch Gerontol Geriatr.

[15] Soares-Miranda L, Siscovick DS, Psaty BM, Longstreth WT Jr, Mozaffarian D (2016). Physical activity and risk of coronary heart disease and stroke in older adults: the cardiovascular health study. Circulation.

[16] Manson JE, Hu FB, Rich-Edwards JW (1999). A prospective study of walking as compared with vigorous exercise in the prevention of coronary heart disease in women. N Engl J Med.

[17] Zaccardi F, Timmins IR, Goldney J (2022). Self-reported walking pace, polygenic risk scores and risk of coronary artery disease in UK biobank. Nutr Metab Cardiovasc Dis.

[18] Tanasescu M, Leitzmann MF, Rimm EB, Willett WC, Stampfer MJ, Hu FB (2002). Exercise type and intensity in relation to coronary heart disease in men. JAMA.

[19] Lee IM, Rexrode KM, Cook NR, Manson JE, Buring JE (2001). Physical activity and coronary heart disease in women: is "no pain, no gain" passé?. JAMA.

[20] Hackett RA, Davies-Kershaw H, Cadar D, Orrell M, Steptoe A (2018). Walking speed, cognitive function, and dementia risk in the English longitudinal study of ageing. J Am Geriatr Soc.

[21] Marino FR, Lessard DM, Saczynski JS (2019). Gait speed and mood, cognition, and quality of life in older adults with atrial fibrillation. J Am Heart Assoc.

[22] Yates T, Zaccardi F, Dhalwani NN (2017). Association of walking pace and handgrip strength with all-cause, cardiovascular, and cancer mortality: a UK Biobank observational study. Eur Heart J.

[23] Fogg BJ (2009). A behavior model for persuasive design. Proceedings of the 4th International Conference on Persuasive Technology.

[24] Duhigg C (2014). The Power of Habit: Why We Do What We Do in Life and Business. Business, by Charles Duhigg. Organ Manag J.

[25] Qazimirsaeed A, Khosravi H, Rafieian M, Mirzahossein H, Forciniti C (2022). Walkability policies in developing countries: what do people need and prefer in Iran?. Sustainability.

